# An open-source slicer for 3D mSLA printing of microfluidic chips

**DOI:** 10.1038/s41598-025-32448-2

**Published:** 2025-12-24

**Authors:** Maria Emmerich, Benjamin Liertz, Robert Wille

**Affiliations:** Technical University of Munich (TUM), Arcisstrasse 21, 80333 Munich, Germany

**Keywords:** Microfluidics, 3D printing, SLA, Slicer, Engineering, Materials science

## Abstract

Microfluidic chips enable high-precision handling and automation of chemical and biological experiments by transporting and manipulating fluids through complex channel networks embedded on-chip. While *polydimethylsiloxane* (PDMS) soft lithography remains the standard for microfluidic device fabrication, it is labor-intensive and requires specialized expertise. *Masked stereolithography* (mSLA) 3D printing offers a rapid, high-resolution alternative, where the 3D chip geometry is fabricated layer-by-layer. These layers are cross-sections of the geometry and stacked on top of each other. However, all existing “off the shelf” slicing processes are optimized for speed and printing outside features rather than the small channels inside microfluidic chips. As a result, the fabrication of these chips still frequently leads to imperfect quality as manifested, e.g., in clogged channels or rough surfaces, which influence the flow through these channels or even render the result unusable. In this work, we propose a novel slicing tool, *OpenSLAice*, that is optimized for 3D printing of microfluidic chips. To this end, we present a slicing method that automatically detects microfluidic features within a microfluidic chip and, then, orients the chip as well as dynamically slices it so that the imperfections in the fabrication of those features are avoided. It rasterizes the sliced cross-layers and, if several parts should be printed at the same time, it automatically arranges them. Finally, a single print exposure calibration is presented to efficiently determine the required exposure times per layer thickness for resin-printer combinations. The modular, open-source tool is available at https://github.com/cda-tum/mmft-openSLAice and allows to pre-process microfluidic chips for mSLA fabrication.

## Introduction

Microfluidic chips allow high-precision control of small fluid volumes, allowing to miniaturize and sometimes even automate complex biological and chemical experiments while reducing reagent use and waste^1,2^. They usually consist of a microfluidic channel network for the transport and manipulation of fluids. This open or closed channel network is embedded in a chip substrate. The development of microfluidic devices stems from the microelectronics industry and has developed into its own distinct discipline, requiring three-dimensional (3D) patterned substrates rather than purely planar features. Traditional manufacturing methods, like *polydimethylsiloxane* (PDMS) soft lithography, remain the fabrication standard for microfluidic chips in industry and academia^1,3^. However, these methods are time and labor-intensive, and often demand specialized expertise.

Instead, additive manufacturing methods like 3D printing offer an alternative for rapid prototyping of microfluidic chips and thereby aid in significantly accelerating their design cycle^4,5^. This makes this method highly attractive for their development and fabrication. But standard 3D printing workflows are optimized for high-speed, and optimizing outside features rather than the small channels inside microfluidic chips. As a result, the channels are often clogged or have rough inner surfaces^6^, which impedes the flow through these channels. By refining the object processing step for printing, also called slicing, the print results can be improved.

### 3D printing via maskSLA

**Fig. 1:**
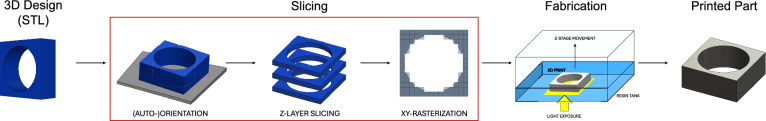
3D printing process.

Additive manufacturing methods, commonly known as 3D printing, build objects layer-by-layer by depositing or solidifying material. Several methods exist, e.g., the widely known fused deposition modeling (FDM), where material is deposited by extruding melted filament via a nozzle, or *stereolithography* (SLA), where a liquid photopolymer resin is cured layer-by-layer via UV exposure. For the fabrication of microfluidic chips, SLA based methods are well-suited, because they allow for higher precision and fewer mechanical inaccuracies due to only z-directional mechanical movement compared to xyz-directional movement for FDM.^7,8^ In the following, we focus on *maskSLA* (mSLA) that is a variant of SLA printing and uses a *liquid crystal display* (LCD) as a dynamic mask in combination with a uniform UV backlight for the light exposure of the resin.

The mSLA 3D printing process is usually conducted in the following four steps (as illustrated in Fig. 1):

1. *3D Design*: Create the design of the desired 3D part in *computer aided design* (CAD) software or via automated design tools ^9–13^.
2. *Slicing*: Import the design into a slicing software that prepares the object for printing. In short, it slices the object into mask images and includes metadata information for printing. For this purpose, “off the shelf” software tools are available, such as *Preform*, *Lychee*, and *ChituBox*.
3. *Fabrication*: Print the desired part. For this, the 3D part is constructed layer-by-layer on a build platform, which is incrementally raised from a vat of liquid resin. The process for each layer is the following:
  - The build platform descends until the distance between the build plate and the transparent *fluorethylen-propylen* (FEP) film at the bottom of the resin vat matches the desired layer height.
  - A short pause called *light-off delay* allows the resin to settle^14^.
  - The masking image is displayed via the LCD and then for a specific *exposure time* the designated regions are cured due to the exposure to the UV backlight.
  - The layer cures from FEP film to build plate, this results in a smooth surface finish on the side directed towards the FEP film, while the inhomogeneous light distribution results in a less polymerized and rougher side towards the build platform.
  - The build plate is lifted to peel the newly cured layer from the FEP film, the *peel force* defines the required force to detach the part from the film^14^.
4. *Printed Part Post-processing*: Remove the part from the build platform, uncured residual resin is washed off and out of it, optionally, the print can be cured to improve material characteristics, and potential scaffolding structures are detached.

Overall, mSLA printing is, in theory, well-suited for the fabrication of microfluidic chips. It operates as a digital photolithography process with programmable masks, enabling high-resolution patterning. The resulting prints typically exhibit smooth xy-surfaces, their resolution defined by the LCD’s pixel size, and a z-resolution defined by the movement of the build platform and specified layer heights^15–18^.

But the slicing strategy, i.e., the way the geometry is sliced into layers, highly influences print quality and subsequently the resulting functionality of the printed microfluidic chip. The inherently layered approach introduces “stair-stepping” artifacts and directly links the print time to the number of layers^6^. Given the importance of the slicing process, it is first described in detail in Section Slicing, and, in Section Limitations of current slicer software, limitations of current slicing tools for printing of microfluidic chips are listed.

### Slicing

Before printing, the 3D part must be converted into a sequence of layer-by-layer instructions^19^. This *slicing* is performed by dedicated software and takes three main inputs:

- *Geometry:* The 3D part, usually defined as a *standard triangle language* or *STereoLithography* (STL) file.
- *Printer specifications:* Build volume, LCD pixel resolution (xy), as well as both z-stage stepping accuracy and height.
- *Resin parameters:* Required exposure time, based on desired layer height, resin, and printer properties^17^.

This leads to a required pre-processing workflow, slicing step, that is usually conducted in four steps (as illustrated in Fig. 1):

1. *(Auto-)orientation:* The part is positioned and oriented on the platform, sometimes this includes scaffolding structures as needed.
2. *Z-layer slicing:* The geometry is sliced at fixed or adaptive intervals along the z-axis (parallel to the build plate). Smaller layers improve feature fidelity in z-direction at the cost of longer print times; adaptive layering balances detail preservation with speed.
3. *XY-rasterization:* Each z-layer interval that was sliced in the previous step is translated into a 2D bitmap image. This xy-rasterization represents the pixels of the printer’s LCD for the light exposure step during printing.
4. *Metadata:* For each layer, associated metadata, like target thickness, exposure time, and delay, are included.

The resulting output of the slicer is a complete build file that includes a series of 2D bitmap images that represent each cross-section at relevant z-heights, as well as the relevant metadata and potential extra required files in a printer-specific format for direct upload to the mSLA printer^17,19^. In the next section, limitations of “off the shelf” slicer software for printing of microfluidic chips are listed.

### Limitations of current slicer software

Several slicers are available for mSLA printing. Three currently prevalent and popular slicers are: *Formlabs PreForm*, *Chitubox*, and *Lychee*. However, while these slicers focus on aesthetic, high-speed prints, while minimizing peel-forces, they are not optimized for printing microfluidic chips with complex internal channel structures. Shortcomings are the following missing features or ones that are limited in their application:

- *(Auto-)orientation* of the 3D part prioritizes minimizing support material and reducing peel forces, but critical aspects such as microfluidic channel alignment or layer-to-layer consistency are not considered. Consequently, channels in microfluidic chips are often tilted relative to the build plane, leading to stair-stepping artifacts and poor surface finishes on critical fluidic structures^15^.
- *Static layer height* is required as an input by the user, forcing a trade-off between fine, slow prints and coarse, fast ones. Even if thin layers are selected, slicers are not aware of microfluidic structures, and layer changes may occur at suboptimal heights, causing geometry loss or distortion of microfluidic features.
- *Static exposure* settings are set for a given layer height, which are not calibrated and usually lead to over-exposure, which results in smaller channel cross-sections or even closed off channels. Additionally, if the layer height is changed, e.g., for changes in layer heights, the exposure time must be recalibrated, which is time-consuming^17,20^.
- *Standardized file formats* are missing for mSLA printing.
- *Closed source slicers and ecosystems* make it impossible to adapt them for specific microfluidic applications or to implement custom calibration procedures.

These limitations often result in rough channel walls or clogged channels^17,20^. While some slicers support individual features, none of them offer a comprehensive solution for mSLA printing of microfluidic chips that would overcome all of these limitations. Furthermore, most established slicers are closed-source, standardized file formats are missing, and even some ecosystems are closed-source, making it impossible to address these limitations directly through custom feature development.

In this work, we address these shortcomings by introducing a slicing algorithm specifically tailored for microfluidic chip fabrication. The algorithm detects microfluidic features within the chip and aligns them to the build plate to maximize geometric fidelity. It supports dynamic layer heights, applying thin layers for microfluidic features and thicker layers for the bulk volume, as well as the required exposure times. For this, we propose *OpenSLAice*, a dedicated open-source slicer for the fabrication of microfluidic chips. In the next section, we introduce the proposed slicing methods for microfluidic devices, and then we evaluate the resulting slicing files and prints. Next, we describe the methods for printing, and finally, Section Conclusion concludes this paper.

## Proposed slicing methods for microfluidic devices

In this section, we describe the features of the proposed slicing method. They are performed in the following order and are comprised of:

1. **Setup:**
  *Initialization*: Printer (build volume, pixel resolution, z-stage movement) and resin (exposure time with regard to layer thickness) parameters are loaded.
  *Geometry Import*: The 3D part (STL) is imported and metadata included.
2. **Core Slicing:**
  *Feature Detection and Orientation*: The microfluidic channel network is detected, and the 3D object is auto-oriented and placed accordingly to optimize channel alignment and reduce stair-step artifacts.
  *Z-Slicing*: Layer heights for slicing are determined, and the object is sliced along the z-axis into 2D planes, based on dynamic or static layer heights.
  *XY-Rasterization*: Each 2D plane is converted into a bitmap mask aligned to the LCD pixel grid.
3. **Output and calibration:**
  *(Optional) Automatic Part Arrangement*: If several objects are printed at the same time, they are arranged on the build platform.
  *Export*: The printer-specific export file, including the printer, resin, and layer data, is created and exported. The modular export logic supports the implementation of additional export formats without modifying the core logic.
  *(Optional) Exposure Calibration*: If necessary, the resin–printer combination can be calibrated using a single print evaluation.

In the following, key features of the core slicing as well as the output and calibration are explained in detail.

### Feature detection and orientation

**Fig. 2:**
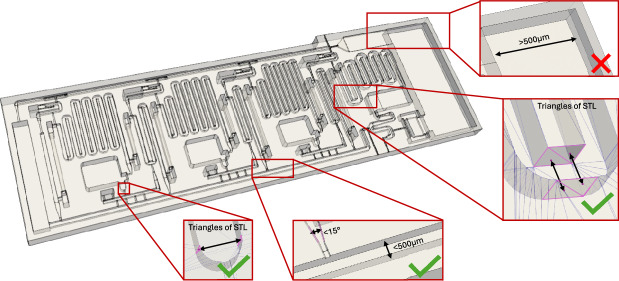
Microfluidic chip geometry with open channels, geometry (STL file) from^21^, exemplary opposing faces and features are magnified. Ones that are detected by the algorithm are marked with a green check mark or a red cross if not.

In microfluidic chips, the channel network is usually aligned within a plane or multiple (connected) planes “stacked” on top of each other. This plane can be aligned with the xy-plane of the printer that has the highest possible resolution to ensure the highest feature fidelity in the microfluidic chip. This alignment will ensure that the channel walls and surfaces have smooth surfaces, while stair-stepping artifacts are minimized, resulting in optimal print quality.

Here, we introduce an automatic orientation algorithm to align the channel network of microfluidic chips with the xy-plane, i.e., the build platform, of a mSLA printer. In contrast to general-purpose slicers, which prioritize reduced peel forces and minimal support generation, this method optimizes the orientation for microfluidic chips to improve print quality.

First, the microfluidic features of the chip need to be identified. For this, opposing faces (more accurately, the triangles that define the faces in STL files, and their centroids) within the 3D STL file are searched for. Identified faces, as illustrated in Fig. 2, need to face each other, and have a distance to each other below a certain threshold (default = 500 $\upmu$m), they can include an angle tolerance of 15° to account for channel geometry variations. Large features that require less accuracy or are not part of the microfluidic network are excluded. Due to the triangle basis of STL files, this also automatically includes rounded features, since they consist of facets of multiple plane triangular faces.

Next, the plane within the 3D geometry with the highest feature count is identified, since this likely corresponds to the feature plane of the microfluidic chip. For this, each opposing face (i.e., its defining triangles and their corner coordinates) that was collected in the previous step was fit to a plane using a *random sample consensus (RANSAC)* algorithm. This algorithm employs sub-sampling to get parameters (i.e., the plane with the highest number of points) from data that contains outliers. For each iteration, a normal was computed based on three vertices, then each vertex in the collection was classified as an inlier if it was within a distance threshold to the plane. The normal with the most inliers is refined via *singular value decomposition* on the inlier set, and the best-fit normal direction is achieved.

Once the feature plane is identified, it can be oriented in parallel to the xy-print plane. Then the part can (optionally) be mirrored to reduce the number of downward-facing features, improving the print fidelity of overhangs.

The algorithm is optimized for highest feature fidelity, disregarding print time (e.g., minimizing z-height) or peel forces. When the microfluidic structure spans multiple planes, no perfect orientation exists, and the algorithm maximizes the number of aligned details. Alternatively, the part can of course also be placed by the user.

### Z-Slicing

**Fig. 3:**
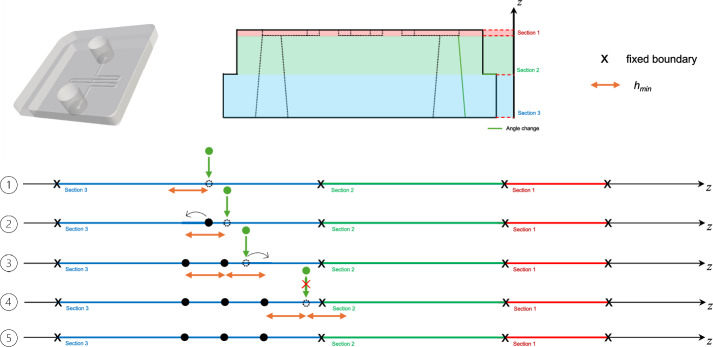
Dynamic z-layer height, geometry (STL file) from^23^.

The mSLA printing process is layer-based, and each layer is cured separately. Large layer heights can result in the loss of fine features, while small layer heights significantly increase print time. Additionally, conventional slicers do not include feature recognition of microfluidic structures. As a result, layer transitions may occur at suboptimal heights, causing geometry loss or distortion. In this section, we introduce a dynamic layer height approach that defines the heights or z-values at which the geometry is sliced.

Dynamic z-layer heights allow to dynamically vary the layer thickness throughout the print. To improve feature fidelity, they should be based on geometry shape and orientation, including layer changes for critical boundaries (e.g., the top or bottom of microfluidic channels). Additionally, fine layer heights reduce stair-stepping artifacts at steep slopes (see also Supplementary Fig. S1), while large layer heights in the bulk volume of the print allow for faster printing times without the loss of feature fidelity^18^.

To determine the z-layer heights, as depicted in Fig. 3, first sections along the z-direction in the print are identified. For this, the algorithm determines section boundaries, i.e., all z-values where (1) the geometry changes shape, (2) orientation, or that (3) define the top or bottom of the previously identified microfluidic network (see Fig. 2 and Section Feature detection and orientation). The start and end point of these sections defines z-values at which the geometry should be sliced. These are set as fixed boundaries, which are prioritized when defining the rest of the required z-values.

In each section, features and steep slopes are detected to determine the best layer heights for each section. This is important to prevent stair-stepping effects and rough surfaces within the microfluidic channels and to accurately define fine features^6^. For sections that include microfluidic features, closing layers are defined that are thicker than the other layers in the section to ensure a robust and structurally stable closing layer. This is also included in the layer height definition. For other sections, features within the sections are identified. Here, slopes connected to microfluidic features are prioritized over steep slopes within each section. To identify these connected surfaces, the surface is simplified to reduce computational overhead, and a *depth-first search (DFS)* is employed that backtracks along a branch, in this case a geometric feature from the microfluidic feature, to find the slopes connected to the microfluidic channel network.

Now, the layer height can be determined for each section that is (1) a multiple of the printer’s resolution $z_{res}$, (2) larger than the minimal height $h_{min}$, and (3) smaller than the printer’s maximum layer height $h_{max}$. For microfluidic feature sections, a stricter maximum height limit (for this printer use case set to $h_{max}=$20 $\upmu$m) is set.

Then, based on the calculated layer height, the z-values for each section are determined and prioritized. The next step, also depicted in Fig. 3 Steps 1 to 5, is a *gap filling algorithm* used to insert the intermediate z-values while enforcing minimal spacing. The minimal spacing is both influenced by (1) the minimal layer thickness $h_{min}$ required by the printer firmware, as well as (2) the fact that the value needs to be a multiple of the maximal resolution of the motorized z-stage $z_{res}$ of the printer hardware. For this, if necessary, the layer heights are rounded to the next resolution step of the z-stage, introducing a maximal error of $z_{res}/2$. If the minimal distance to the previous value is below $h_{min}$, the lower priority value is shifted or dropped. Finally, an additional offset is added to prevent the erroneous inclusion of terminal surfaces at slice boundaries.

Alternatively, a forced layer height ($>h_{min}$) can be specified by the user.

Overall, the described slicing method detects critical microfluidic features, specifically channel boundaries, steep slopes, and surface orientations, resulting in a dynamic z-layer height. This improves print quality and efficiency. Additionally, a list of z-values at which the 3D part should be cut is defined, these cuts or 2D cross-sections are defined in the next section.

### XY-rasterization

**Fig. 4:**
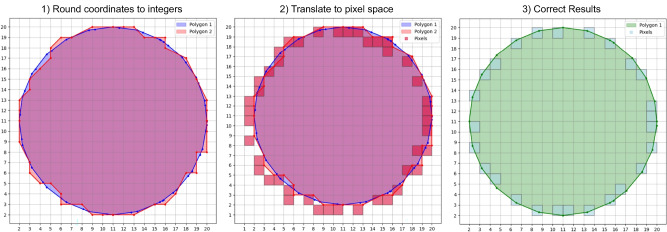
XY-rasterization.

After the slicing heights are defined (the z-values defined in the previous section), a slice or cross-section of the 3D part is generated. This cross-section yields 2D path objects consisting of 2D point sections at each z-value. These are then rasterized and transformed into a 2D black and white bitmap image by converting the contour from millimeters to pixel space. The pixelated 2D cross-section will serve as input for the LCD mask of the printer to determine where UV light will cure the resin. White pixels represent solid regions, black pixels remain unexposed. The required steps for the LCD mask generation are:

- generation of intersections,
- rasterized image output, and
- improvement of the rasterized images.

In Fig. 4, the rasterization of each cross-section is depicted. The proposed rasterization method is based on the *trimesh* library with adaptations to improve the result. For this, the rasterization method of the *rasterio* library is employed, which preserves dimensional accuracy by mapping each pixel directly according to its physical location in the pixel space. Next, see also Fig. 4, introduced errors due to edge touching pixels are fixed by, at first, using Bresenham’s line algorithm, only selecting pixels with a center within the polygon contour^22^.

Optionally, the print fidelity can be enhanced by employing anti-aliasing. For this, each slice is rendered at a higher resolution and then downsampled to the target printer resolution. Because in mSLA printing, gray scale values below a certain threshold $min_{aa}$ cannot reliably cure the resin, pixels with a value below $min_{aa}/2$ are set to zero (black) and to $min_{aa}$ if they are above half its value. This ensures that the exposure for all pixels is high enough to cure the resin.

Once all cross-sections are rasterized, they are fit onto the build plate at their designated position and combined to a single stack of binary mask images, ready for export.

**Fig. 5:**
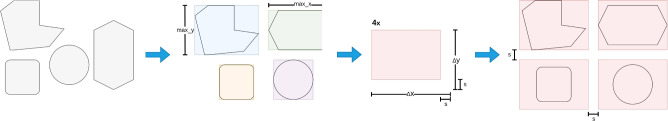
Automatic part arrangement.

### Automatic part arrangement

When 3D printing a part, more than one object can be printed at the same time, if they fit on the available space on the build platform. For this, the parts are arranged automatically. In Fig. 5, the arrangement process is visualized. First, a bounding box is used to define the shape of the part and prevent overlaps, then a minimal spacing distance between the modules is added, and they are placed using a center-out grid-filling approach.

### Export file(s)

There is no standardized version of printer-specific files for mSLA printers. Each printer manufacturer defines its own proprietary file format, often without providing any formal documentation.

The proposed software tool creates files for the *Anycubic Photon Mono 4 Ultra*, and its *.pm4u* import file format, as well as beta export files for *Elegoo* resin printers, like the *Mars 5 Ultra*, using the *.goo* format. The file export logic is modular to explicitly make it easier to support additional formats to be able to extend the code without modifying the core logic. Here, the *.goo* export serves primarily as an example of how the code can be extended to support additional printer formats. In the following, we focus on the *.pm4u* export and validate its output.

### Exposure calibration

**Fig. 6:**
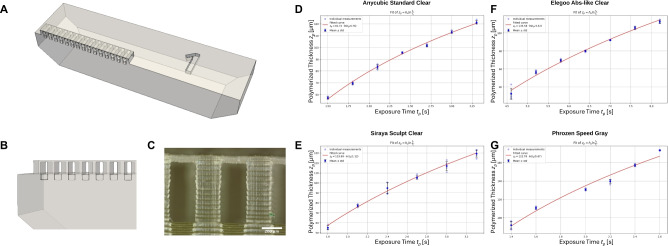
Exposure Calibration. **A, B** show the proposed Geometry Design, **C** shows an exemplary optical microscope picture of the membrane, **D-G** show the membrane layer thickness for different exposure times, error bars show standard deviation (n = 4) for each data point. Used resins: **D** Anycubic Standard Clear, **E** Siraya Sculpt Clear, **F** Elegoo Abs-like Clear, and **G** Phrozen Speed Gray.

Each combination of resin, printer, and layer height requires a specific exposure time to ensure dimensional accuracy and a good surface finish. If the exposure time is too long, the print quality and accuracy decrease, including the closing off of microfluidic channels, while deficient exposure time impedes correct attachment of the new layer^14^. The exposure time is an input parameter for the printer and should therefore be supplied as part of the slicer output, subsequently the proposed *OpenSLAice* software requires the exposure time as function of layer height. Unfortunately, this data is often not available for the desired resin-printer specification or combination. When changing any of the aforementioned parameters (resin, printer, or layer height), the calibration process must be repeated, which slows down prototyping and makes arbitrary exposure times for dynamic layer heights impractical. To be able to utilize correct exposure times, we propose a single print exposure calibration.

The exposure time is a function of the layer height, the resin’s optical properties, and the printer’s UV power. To calculate the critical exposure time, a model, based on the *Beer-Lambert law*, can be utilized^17^

1 $$\begin{aligned} z_p = h_a ln \frac{t_p}{T_c}, \end{aligned}$$

where the relation between polymerization depth $z_p$, exposure time $t_p$ is defined by the resin-specific variables $h_a$ and $T_c$, where the latter is also influenced by the printer’s light intensity. Specifically, $h_a$ and $T_c$ are then used as an input for *OpenSLAice*. To calculate these required variables, multiple measurements of polymerization depth $z_p$ per exposure time $t_p$ are required. For this, a dedicated 3D part that is depicted in Fig. 6A was designed that includes a single-layer membrane to measure $z_p$. Multiple versions of this part, with varying heights, can be printed at the same time, allowing to define different final membrane exposure times and subsequently thicknesses depending on how deep the UV light polymerizes the resin. In Fig. 6B, the column structure of the designed 3D geometry that is closed by a membrane is depicted, and in 6C, a microscope picture of this is depicted. In Panel 6D to G, the resulting membrane thicknesses for different exposure times that were measured using an optical microscope are plotted for four different resins. The applied model then allows to calculate the required exposure time for desired heights, for the used resin printer combination. Additionally, the irradiance at the printer screen can be directly measured using an optical power sensor to be able to reuse the calibration data for any other printer, however, we will not go into depth on this method here.

Overall, the proposed method detects microfluidic features, automatically orients the 3D part, dynamically slices it based on those features, and rasterizes each resulting cross-section to achieve the best possible accuracy and surface finish. When multiple parts should be printed, they can be automatically arranged on the build platform of the printer. We also include a single print exposure calibration to determine optimal exposure times for different layer heights. The resulting open-source tool *OpenSLAice* for 3D mSLA printing of microfluidic chips is available at https://github.com/cda-tum/mmft-openSLAice. In the next section, the resulting tool is evaluated by investigating the auto-orientation of the print, the resulting z-layer heights after slicing, the xy-accuracy of the generated cross-sections, and the resulting prints.

## Evaluation

The proposed *OpenSLAice* tool prepares and slices microfluidic chip geometries for mSLA printing. In the following, the designated auto-orientation of the microfluidic channel network, dynamic z-layer slicing with regard to microfluidic features of the digital results, as well as the xy-rasterization of each cross-section and resulting prints are evaluated. Additionally, we compared the tool to three mSLA slicers *Lychee Slicer* (https://mango3d.io/download-lychee-slicer, v7.3.2), *ChituBox Basic* (https://www.chitubox.com/en/download/chitubox-free, v2), and *PreForm* (https://formlabs.com/software/preform/, v3.33.2). In the following, these slicers are listed without their version specification.

**Fig. 7:**
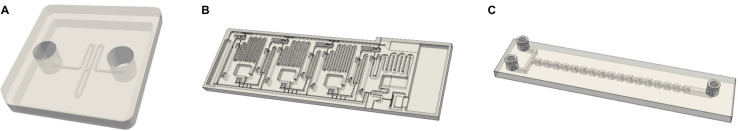
Examples of microfluidic chips to evaluate the proposed slicing tool. **A** *spheroid-trap*, an open channel chip, geometry (STL file) from^23,24^, **B** *ELISA* chip, an open channel chip for biological analyses, geometry (STL file) from^21^, and **C** *mixer*, a closed channel microfluidic chip for mixing of two fluids, geometry (STL file) from^21^.

For the evaluation, three exemplary microfluidic geometries from literature were selected. In Fig. 7, these geometries are depicted. The STL files are available in the GitHub repository and via the respective references. Specifically,

- an open channel design that includes a channel loop and a fluidic trap to trap spheroids (cell aggregates) on-chip for biological experiments, from^23,24^, in the following referred to as *spheroid-trap*,
- an open channel chip for a so-called ELISA assay, a biological analysis method, from^21^, referred to as *ELISA* chip, and finally,
- a closed channel design of a microfluidic mixer, from^21^, referred to as *mixer*.

### Feature detection and orientation

**Fig. 8:**
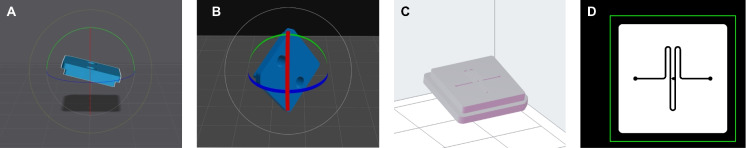
Automatic part arrangement of the *spheroid-trap* microfluidic chip, geometry (STL file) from^23^ in **A** *Lychee* slightly tilted, **B** *ChituBox* highly tilted, **C** *PreForm* slightly tilted, visualized via the respective user interface of the tools, **D** the proposed tool *OpenSLAice* flat on the build platform visualized via *UVtools*^25^. **A** to **C** are nondeterministic orientations, i.e., the result changes with each re-orientation.

The auto orientation feature of the proposed *OpenSLAice* tool is compared to the outputs of three popular slicers *Lychee*, *ChituBox*, and *PreForm*. The auto-orientation in all other slicers is nondeterministic, i.e., the orientation of the part differs each time. It focuses on minimizing peel forces, or for *Preform* on preventing so-called “cupping” effects, where air is trapped inside of a section of the part, that usually has a cup-like geometry, and due to the outwards movement (out of the resin vat) the part is damaged. This risk is very small for small cups or thicker walls, and therefore not applicable to most microfluidic chips. For ten auto-orientations per tool, each one places the part at an angle. In Fig. 8, an example of this angled geometry for each tool is depicted in Panels 8A to C, in Panel 8D, the result of *OpenSLAice* is depicted, where the microfluidic channel network is aligned with the build platform. The aligned network will be printed at a better resolution, since stair-stepping effects are minimized and the greater resolution of the printer’s xy-plane is utilized.

### Z-slicing

**Fig. 9:**
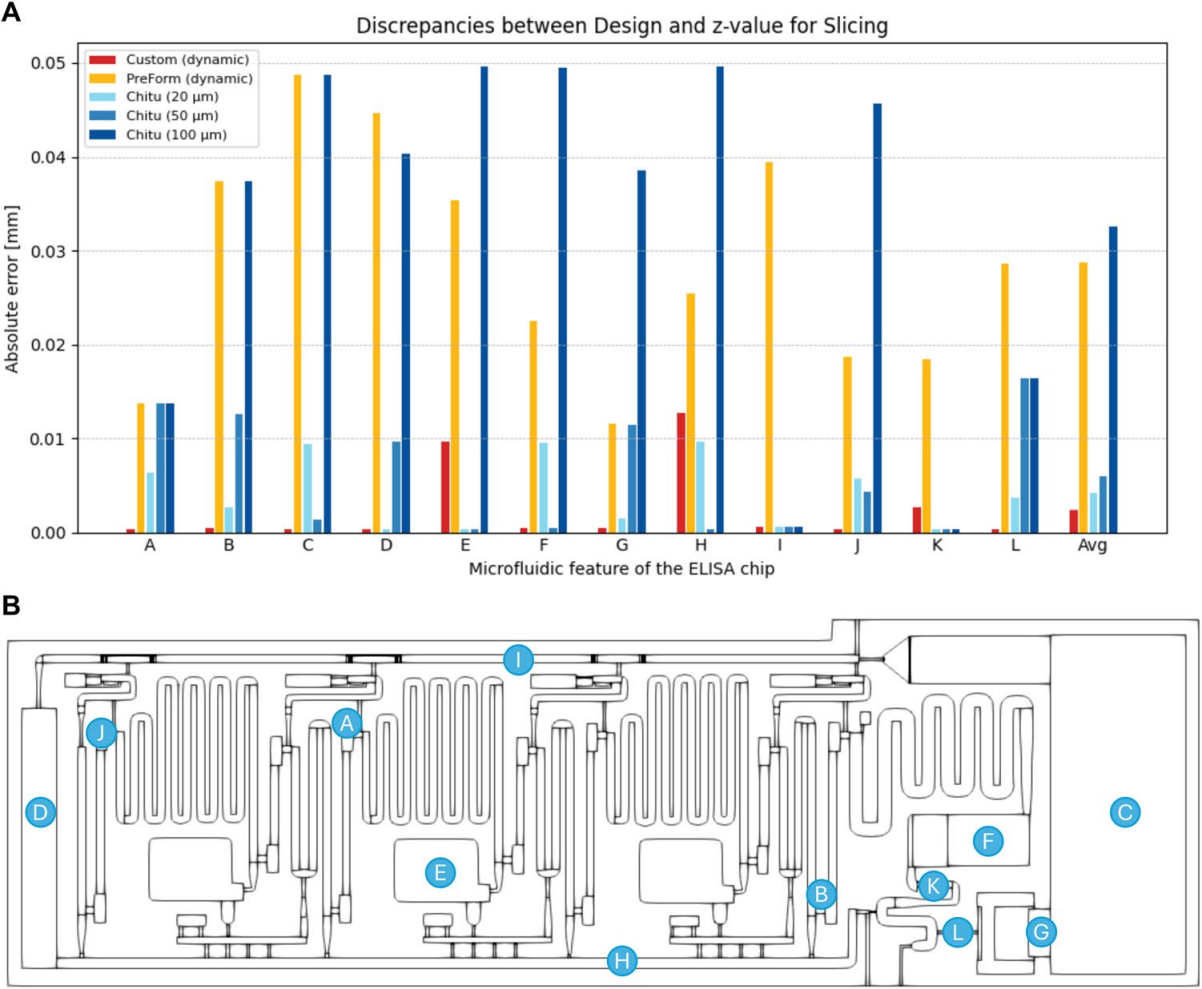
Z-Layer Evaluation of different slicers, including dynamic slicing by the custom method and *PreForm*, as well as static layer heights of 20, 50, and 100  $\upmu$m by *ChituBox*. **A** shows the absolute error between the design features respectively and the z-value for slicing determined by the tools in millimeters and **B** the microfluidic features that were selected for the evaluation of z-layer values on the *ELISA* chip, geometry (STL file) from^21^.

To determine the precision in z-direction, the resulting z-values each slicer calculates were analyzed. For this, the *ELISA* chip was selected due to its numerous distinct z-steps. The chip design was analyzed, and twelve distinct feature heights were measured using *Onshape*. Next, the chip was sliced using three slicers, (1) dynamic slicing using the proposed method, (2) dynamic slicing using *Preform*, and (3) static layer heights from *ChituBox* at 20, 50, and 100  $\upmu$m, here, the resulting layer heights were identical to *Lychee*. The slicer output was measured by extracting the layer heights from the sliced model using *UVTools*^25^, or in the case of *PreForm*, analyzing the slices in the user interface of the tool.

In Fig. 9, the desired design was compared to the layer heights after slicing of each tool. The features that were analyzed are marked on the chip, and the graph shows the error in height at each feature.

Overall, the custom slicer achieves the lowest $z$-axis error of the three slicing approaches, demonstrating superior fidelity compared to the dynamic slicing of *PreForm*. The number of slices of each result is 133 for the custom method, 53 for *PreForm*, and for *ChituBox* and *Lychee*: 139 at 20 $\upmu$m, 55 at 50 $\upmu$m, and 27 at 100 $\upmu$m. Since print time scales with the number of slices, the 20 $\upmu$m static slicing method has the highest print time, followed closely by the custom slicing method, which also produces very thin layers of about 10 $\upmu$m near critical features. Using thin layers helps to improve the wall structure and reduce stair-stepping artifacts (see Supplementary Fig. S1). While the average error of static 20 $\upmu$m setting of *ChituBox* is closest to the tool, it still requires slightly more layer heights and subsequently slightly longer printing times. When the bulk volume is larger, e.g., for the spheroid chip, the difference in layers becomes more significant, the custom method generates only 39 layers, compared to 149 layers with *ChituBox* at 20 $\upmu$m.

### XY-rasterization

To evaluate the xy-rasterization capability of the proposed slicing tool, first, a mask layer of the tool was compared to the resulting mask layers of other slicers, specifically *ChituBox* and *Lychee*. This way, the accuracy of features, e.g., edges and corners of channels that lie within these planes, can be investigated. Additionally, the xy-resolution of the three example chips depicted in Fig. 7 was analyzed, both of the mask and of the resulting print.

**Fig. 10:**
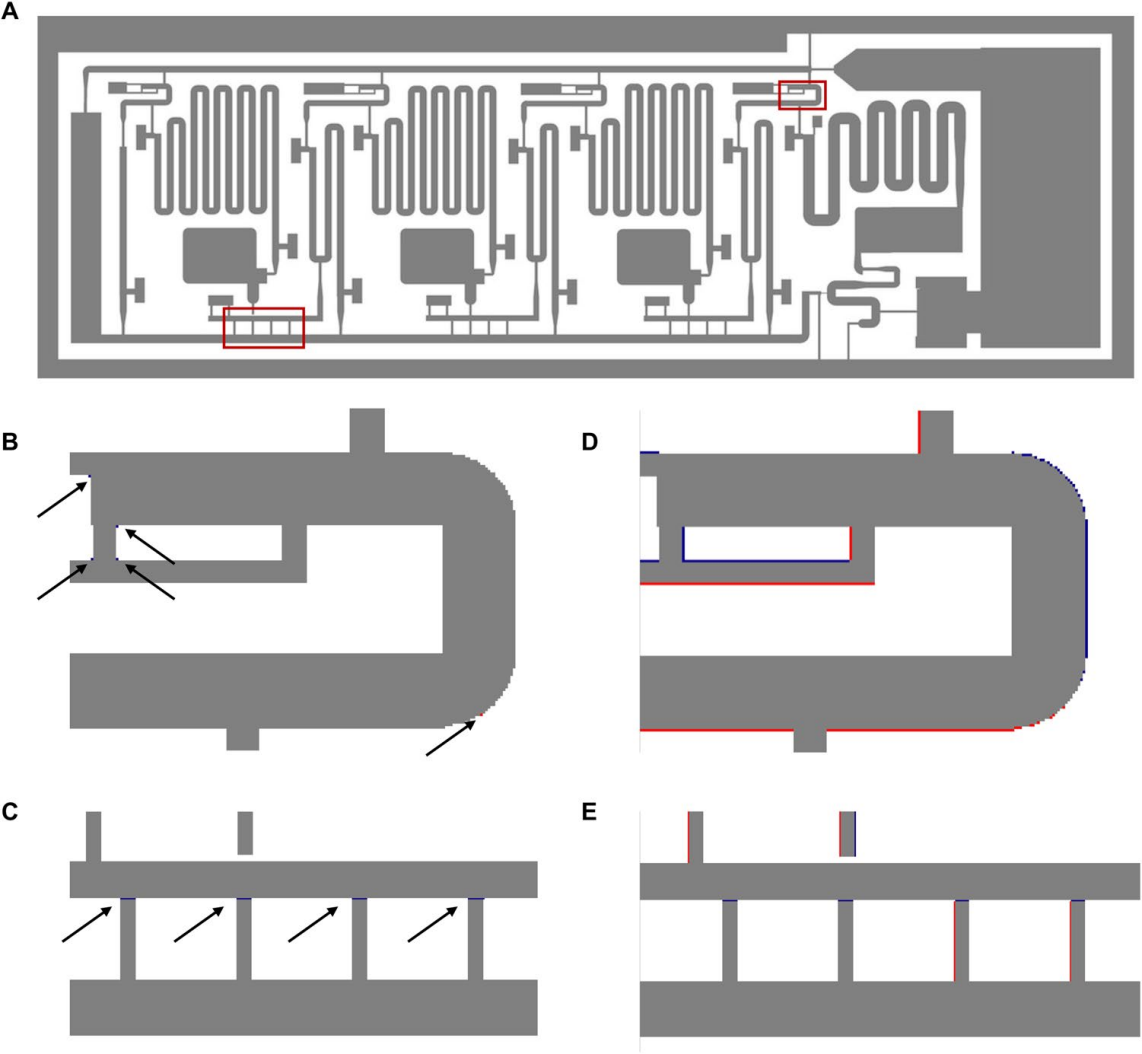
Comparison of the resulting xy-rasterization using the *ELISA* chip, geometry (STL file) from^21^ between the proposed slicing tool *OpenSLAice* and **B, C** *Chitubox* or **D, E** *Lychee*. Images generated using *Python*. Grey pixels are areas of the mask that will not be exposed, white pixels will be exposed in all three tools. Blue pixels are not exposed in the compared tools, but not in the proposed tool, red pixels are exposed in the proposed tool but not in the other tools.

**Fig. 11:**
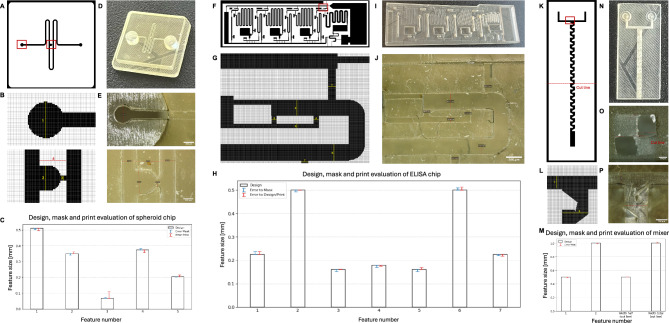
XY-feature analysis of the three exemplary chips.**A-E** shows the *spheroid-trap* chip, **F-J** the *ELISA* chip, and **K-P** the microfluidic *mixer*. **A, F, K** depict one xy-cross-section mask layer, and **B, G, L** zoomed in microfluidic features of the respective exemplary chips, masks are visualized using *UV tools*,^25^. **D, I, N** depict photographs of the three 3D printed chips, **E, J, O, P** depict magnified features of the chips. **O** shows the cut line of the *mixer*. **C, H, M** depict the design goal of each selected feature of the three chips, and the error of the mask (blue) and print (red) in mm.

In Fig. 10, a mask image of a complex microfluidic network, specifically the *ELISA* chip, was compared between the three slicers: *Chitubox*, *Lychee*, and the proposed tool *OpenSLAice*. The resulting masks are quite similar. However, there is a shift in the mask results by the *Lychee* slicer, and some pixels at rounded corners are different between all three slicers. One noticeable change is that in Fig. 10C and E, in the compared slicers, a line of pixels is not exposed that exists in the initial design. This line is (correctly) exposed in the mask of the proposed tool, showcasing the high fidelity of the proposed xy-rasterization approach. However, the line is too small to appear in printed parts of the used printer and its mask resolution, and might be the result of a design error. No comparison to *PreForm* was possible, as the rasterized mask layers can not be visualized or exported from the tool.

Additionally, the xy-accuracy of the proposed tool was analyzed by comparing the desired design to the resulting mask images and to 3D printed versions of the slicing results. In Fig. 11, for each of the three example chips used for the evaluation, first mask images were generated by the proposed slicer (see Fig. 11A, F, and K), and the resulting files were printed. Photos of the printed chips are depicted in Fig. 11D, I, and M. Then, key microfluidic features were measured respectively to evaluate accuracy. In Fig. 11, first the features were measured within the masks, then analyzed using an optical microscope of the printed chip, and lastly the errors of mask (blue error bars) and print (red error bars) were compared to the initial design goal, see Fig. 11E, H, and M. All mask images were generated for and printed with the Anycubic Mono 4 Ultra printer, which has a pixel size of 17 $\upmu$m. At this resolution, light scattering effects are significant enough to smooth out stair-stepping artifacts in the xy-plane that are introduced by the rasterization of the layer images, leading to smooth edges in the xy-plane even without anti-aliasing (see Fig. 11 and Supplementary Fig. S2). The errors of both the mask and the print are very small, especially compared to the design goal, and can be explained by the pixel size of the printer and very small fabrication inaccuracies.

Overall, the xy-resolution of the resulting masks is comparable to other slicers and even represents tiny features better than the compared slicers. It delivers accurate mask layers for the printing process, and this way supports accurate prints with smooth edges that can represent desired corner shapes, if the printer hardware allows.

The evaluation demonstrates that the proposed method reliably detects microfluidic features in three representative chip designs and uses this information to correctly auto-orient the part. Compared to existing slicers, our approach enables more precise dynamic slicing by aligning layer transitions with critical microfluidic geometries. The resulting xy-rasterization produces high-resolution bitmap masks that are comparable to, and in some cases more detailed than, those generated by established slicers. This results in a slicer that is well-suited to the specific requirements of microfluidic chip fabrication.

## Methods

All figures of 3D parts are visualized using *Paraview* (https://www.paraview.org/, v5.11.0) ^26^, unless otherwise specified. The digital evaluations were deterministic, i.e., the same result is obtained for each run and subsequently a single evaluation (n=1) was performed.

### 3D printing

All test parts were printed using an *Anycubic Photon Mono 4 Ultra* printer. A standard *fep* vat from the Mono 2 was used instead of the default ACF film to avoid the diffusion problems associated with the latter. The LCD mask has a resolution of 9024 $\times$ 5120 pixels, with a pixel size of $17 \times$17 $\upmu$m. The maximum build height is 165 mm, with a z-stage resolution of 1 $\upmu$m and the minimal layer height of 10 $\upmu$m.

For bridge spans, 40 $\upmu$m was selected as a minimum layer height to maintain structural integrity (this is included in the software layer height calculation). Features aligned with the xy-axis should be at least four pixels wide. These values represent practical fabrication limits when using the described hardware and resins. The following movement parameters were used for all prints:

- bottom lift height = 8 mm
- bottom lift speed = 1.5 mm/s, bottom lift retract speed = 3 mm/s
- normal lift height = 6 mm
- normal lift speed = 2 mm/s, normal lift retract speed = 3 mm/s
- light off delay = 2 s

All prints were carried out at room temperature using the following resins:

- Phrozen Speed Gray
- Anycubic Standard Clear
- Siraya Tech Sculpt Clear
- Elegoo ABS-Like (v1) Clear

Post-processing included cleaning in 99.9% isopropanol (2min soak + 1min agitation in fresh bath) followed by targeted rinsing using a syringe. Parts were dried with compressed air and cured for 3min in an *Anycubic Wash & Cure Plus*.

Each print was printed one time (n=1) unless otherwise specified.

### Print analysis

Images for dimensional measurements were captured using a *Leica DVM6* digital microscope equipped with a polarization filter to minimize reflections while preserving surface detail for focus alignment.

Illumination was adjusted to RL 60–90, with coaxial light (CXI) turned off. Images were captured with the *Leica Application Suite X* (https://www.leica-microsystems.com/products/microscope-software/p/leica-las-x-ls/, v5.3.0.26130) at 2 MP resolution.

Measurements were annotated using *QuPath* (https://qupath.github.io/, v0.5.1) via line tools; These annotations were exported and processed using Python for quantitative analysis.

## Conclusion

This work presented an open-source slicing tool for mSLA printing of microfluidic chips. The proposed method automatically detects microfluidic features, based on these, the chip is oriented, and the cross-sections are sliced to generate the best printing results. The method also includes xy-rasterization and automatic part arrangement, should several prints be printed at the same time. Additionally, we propose a single-print exposure calibration approach to measure the connection between exposure time and layer thickness for any resin-printer combination. The orientation, z-slicing, and xy-rasterization were evaluated by comparing the results to currently prevalent and popular slicing tools, analyzing the digital results of the slicer, as well as analyzing the resulting 3D printed chips. The results clearly show that the proposed tool is an efficient and optimized way to preprocess microfluidic chips for mSLA printing. The tool is available as an open-source software package at https://github.com/cda-tum/mmft-openSLAice.

Future progress in the field could further enhance the applicability of the tool. In particular, broader adoption of standardized input file formats for mSLA printers and consistent resin classification would enable more seamless integration across systems. For now, the tool can be extended to accommodate additional export formats if needed. Other promising directions for future development include incorporating technological advances like multi-material applications or dynamic tank movement.

## Supplementary Information

Supplementary Information.

## Data Availability

The open-source tool and all associated STLs are publicly available at https://github.com/cda-tum/mmft-openSLAice, as part of the Munich Microfluidics Toolkit (MMFT)^27^
