# Supplementary material for: An open-source slicer for 3D mSLA printing of microfluidic chips
Source: Sci Rep. 2025 Dec 24;15:44416. doi: 10.1038/s41598-025-32448-2 (PMC12738676; doi:10.1038/s41598-025-32448-2)
Supplement: Supplementary file 1 — Supplementary Information. [file 41598_2025_32448_MOESM1_ESM.pdf]

Supplementary Material

An Open-Source Slicer for 3D mSLA Printing of Microfluidic Chips

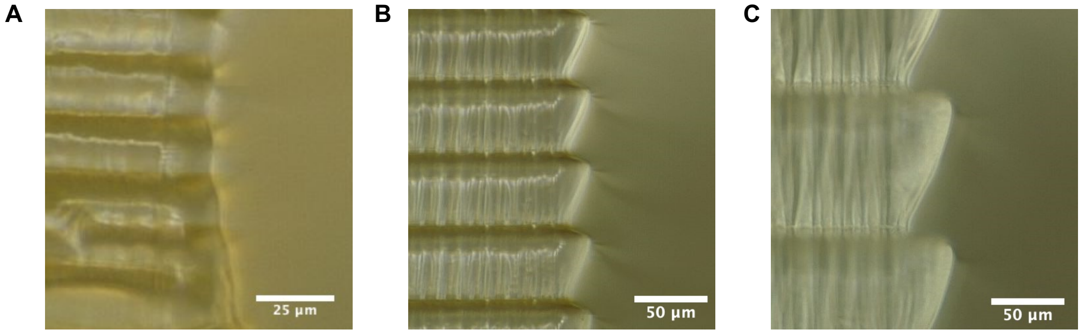

Fig. S 1: Microscopy image of the side of 3D printed parts, with **A** 20 µm, **B** 50 µm, and **C** 100 µm thick layers. They show the distinct layers and stair-stepping artifacts on the wall.

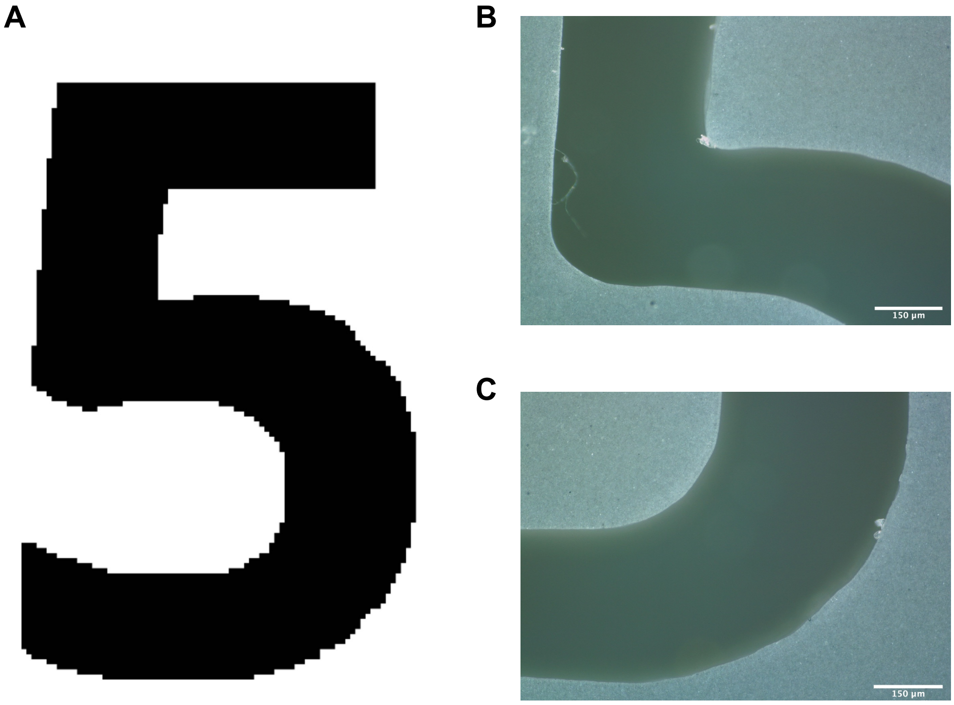

Fig. S 2: XY-resolution of mask and printed parts. **A** mask layer with an xy-resolution of 17 µm, **B**,**C** microscopy images of the top-side of 3D printed parts.
